# 1738. Cost-Effectiveness Analysis of Infant Pneumococcal Vaccination with the 20-Valent Pneumococcal Conjugate in the US

**DOI:** 10.1093/ofid/ofad500.1569

**Published:** 2023-11-27

**Authors:** Mark Rozenbaum, Liping Huang, Johnna Perdrizet, Alejandro D Cane, Adriano Arguedas, Kyla Hayford, Maria J Tort, Ruth Chapman, Vincenza Snow, Erica Chilson, Ray Farkouh, Desmond Dillon-Murphy

**Affiliations:** Pfizer Inc., Randstad, Noord-Holland, Netherlands; Pfizer Inc, Collegeville, Pennsylvania; Pfizer Inc, Collegeville, Pennsylvania; Pfizer, Collegeville, Pennsylvania; Pfizer, Collegeville, Pennsylvania; Pfizer, Collegeville, Pennsylvania; Pfizer Inc, Collegeville, Pennsylvania; Evidera, Inc, London, England, United Kingdom; Pfizer Vaccines, Collegeville, PA; Pfizer, Collegeville, Pennsylvania; Pfizer, Inc., New York, New York; Evidera PPD, London, England, United Kingdom

## Abstract

**Background:**

As of March 2023, two pneumococcal conjugate vaccines, 13- (PCV13) and 15- (PCV15) valent formulations, are recommended for US infants under a 3+1 schedule. PCV20 was approved by the FDA in June of 2021 for adults ≥18 years and most recently in April 2023 for children < 18 years. This study evaluates the health and economic impact of vaccinating US infants with a new expanded valency PCV20 formulation.

**Methods:**

A population-based, multi cohort, decision-analytic Markov model was developed to estimate the public health impact and cost-effectiveness of PCV20 from both a societal (base-case) and health care payer perspective over a 10-year time horizon. Epidemiological data were based on published studies and unpublished Active Bacterial Core Surveillance System (ABCs) data. Vaccine effectiveness estimates were based on PCV13 effectiveness and PCV7 efficacy studies. Indirect impact assumptions were based on observational studies which showed gradual disease reduction in all ages after the introduction of PCV7/13. Costs and disutilities were based on published data. PCV20 was compared against both PCV13 and PCV15 in separate scenarios.

**Results:**

Replacing PCV13 with PCV20 in infants has the potential to avert over 55,000 invasive pneumococcal disease (IPD) cases, 2.5 million pneumonia cases, 5.4 million otitis media (OM) cases, and 19,000 deaths across all ages over a 10-year time horizon. This corresponds to a net gain of 270,000 life years or 515,000 QALYs. Acquisition costs of PCV20 were offset by monetary savings from averted cases resulting in $20.6 billion net saving. The same trend was observed when comparing PCV20 versus PCV15 with a net gain of 280,000 QALYs and $10.3 billion of net savings. A large proportion of the avoided costs and cases were attributable to indirect effects in unvaccinated adults and elderly. From a health-care perspective PCV20 was also the dominant strategy compared with both PCV13 and PCV15.

**Conclusion:**

Infant vaccination with PCV20 was estimated to be cost-saving compared with both PCV13 and PCV15. This study demonstrated implementation of a routine infant vaccination program with PCV20 will substantially reduce pneumococcal disease burden and save health-care costs.

**Disclosures:**

**Mark Rozenbaum, Ph.D., M.B.A.**, Pfizer: Stocks/Bonds **Liping Huang, MD, MA, MS**, Pfizer Inc.: Stocks/Bonds **Johnna Perdrizet, MPH**, Pfizer Inc: Stocks/Bonds **Alejandro D. Cane, MD, PhD**, Pfizer: Stocks/Bonds **Adriano Arguedas, Medical director**, Pfizer: Emplyee|Pfizer: Stocks/Bonds **Kyla hayford, PhD**, Pfizer: Employee|Pfizer: Ownership Interest|Pfizer: Stocks/Bonds **Maria J Tort, PhD**, Pfizer Inc: Stocks/Bonds **Ruth Chapman, MSc, PhD**, Pfizer: Advisor/Consultant **Vincenza Snow, MD**, Pfizer Vaccines: employee|Pfizer Vaccines: Stocks/Bonds **Erica Chilson, PharmD**, Pfizer, Inc: Ownership Interest|Pfizer, Inc: Stocks/Bonds **Ray Farkouh, PhD**, Pfizer: Stocks/Bonds **Desmond Dillon-Murphy, MSc, PhD**, Pfizer: Advisor/Consultant

